# Folic acid derived-P5779 mimetics regulate DAMP-mediated inflammation through disruption of HMGB1:TLR4:MD-2 axes

**DOI:** 10.1371/journal.pone.0193028

**Published:** 2018-02-15

**Authors:** Shan Sun, Mingzhu He, Yongjun Wang, Huan Yang, Yousef Al-Abed

**Affiliations:** 1 Center for Molecular Innovation, The Feinstein Institute for Medical Research, Manhasset, New York, United States of America; 2 Department of Biomedical Science, The Feinstein Institute for Medical Research, Manhasset, New York, United States of America; Katholieke Universiteit Leuven Rega Institute for Medical Research, BELGIUM

## Abstract

High mobility group box 1 (HMGB1) is a damage-associated molecular pattern (DAMP) protein that mediates inflammatory responses after infection or injury. Previously, we reported a peptide inhibitor of HMGB1 (P5779) that acts by directly interrupting HMGB1/MD-2 binding. Here, fingerprint similarity search and docking studies suggest folic acid derived-drugs function as P5779 mimetopes. Molecular dynamic (MD) simulation studies demonstrate that folic acid mimics the binding of P5779 at the TLR4 and MD-2 intersection. In surface plasmon resonance (SPR) studies, these drugs showed direct binding to TLR4/MD-2 but not HMGB1. Furthermore, these P5779 mimetopes inhibit HMGB1 and MD-2 binding and suppress HMGB1-induced TNF release in human macrophages in the nanomolar range. We assert from our findings that their demonstrated anti-inflammatory effects may be working through TLR4-dependent signaling.

## Introduction

High mobility group box 1 (HMGB1) protein has dual roles, DNA binding and cytokine-like inflammatory activity [1]. It is an essential and ubiquitous DNA architectural factor that regulates cellular processes, including transcription, chromatin remodeling, recombination and DNA repair by modulating chromosomal DNA structure. In response to infections and injuries, HMGB1 is secreted from activated immune cells or passively released from injured cells. Excessive extracellular HMGB1 contributes to the pathogenesis of infection- and injury-elicited inflammatory diseases. The disulfide HMGB1 activates immune cells to produce cytokines/chemokines via Toll-like receptor 4 (TLR4), receptor for advanced glycation endproducts (RAGE), or other receptors [2]. The extracellular HMGB1 has been established as a pathogenic mediator of both infection- and injury-elicited inflammatory diseases.

Previously we reported that HMGB1 induces inflammatory responses via the TLR4 signaling pathway and that interaction with the TLR4/MD-2 complex requires the disulfide HMGB1. By screening the HMGB1 peptide libraries, a tetramer designated P5779 (FSSE) was identified as a specific MD-2 antagonist that inhibits HMGB1/MD-2 interaction and thereby TLR4 signaling [3]. Furthermore, P5779 protected mice against hepatic ischemia/reperfusion injury, chemical toxicity and sepsis. Our current study takes an *in silico* insight into the interaction of P5779, as well as the inactive scramble peptides, and the TLR4/MD-2 complex, to explore the mechanism of action. To overcome the poor pharmacokinetic profiles of P5779, we applied a virtual fingerprint similarity search on Drugbank database (version 4.3) to identify P5779 mimicking small molecule drugs. A set of pteroglutamic acid analogue drugs, such as folic acid, methotrexate (MTX), etc., shared similar binding poses with P5779 on the TLR4/MD-2 complex. Direct binding studies using SPR, further illustrated these molecules bind to MD-2 and inhibit the HMGB1/MD-2 interaction. Finally, *in vitro* studies demonstrated the ability of these molecules to inhibit HMGB1-induced TNF release in human-derived macrophages with higher potency compared with P5779. Based on our results, we propose that folic acid (an FDA approved supplement) and related analogue drugs with good pharmacokinetic profiles, can be used as P5779 mimetics to attenuate DAMP-mediated inflammation.

## Materials and methods

### Reagents

P5779 peptide was custom-made from Genemed Synthesis, Inc. with more than 95% purity as determined by HPLC. Recombinant HMGB1 was expressed in E. *coli* and purified to homogeneity as reported previously[4]. Folic acid and analogues were purchased from Sigma-Aldrich.

### Docking study

The protein preparation wizard in Maestro (Schrödinger, LLC, New York, NY, 2016) [5]was used to prepare the TLR4/MD-2 complex (PDB: 3FXI, chain A and C) structure imported from the Protein Data Bank. LPS, ions and water molecules were removed from the structure. A receptor grid, which can accommodate ligands with length up to 20Å, was generated and located in the center to cover the entire MD-2 structure by GLIDE (Schrödinger, LLC, New York, NY, 2016) [6–8]. Compounds were constructed with 2D Sketcher or imported from the DrugBank database (version 4.3). All ligands underwent energy minimization and ligand preparation using LigPrep. Extra precision (XP) mode of Glide dock [7, 8] was executed using a flexible ligand and a rigid receptor routine. At most, 10 docking poses were obtained from each molecule.

### Fingerprint-based similarity search

We conducted a P5779 fingerprint-based similarity search against the DrugBank database (version 4.3) [9]. The constructed P5779 structure was used as the template. A total of 1,982 FDA approved or investigational drugs from the database was prepared by energy minimization using MMFF94x forcefield. Three fingerprint types, MACC structure key (BIT-MACC), shape (ESsape3D) and pharmacophore atom triangle (piDAPH3), were calculated against each molecule in the database. The Tanimoto coefficient [10] was applied to score compound similarity with BIT-MACCS. For ESshape3D and phDAPH3 fingerprints, the similarity scores are calculated as the inverse of the distances between two corresponding fingerprints [11]. All the hits were then subjected to docking and MM-GBSA refinement in comparison with P5779. Molecules with docking scores better than P5779 were considered as P5779 mimetic hits.

### MD simulation

The ligand-protein complex from the docking study was subjected to MD simulation performed using Desmond program (Schrödinger, LLC, New York, NY, 2016) [12, 13]. The complex was solvated with the TIP3P water model [14] in a 10Å ×10Å×10Å orthorhombic box. The simulation was carried out with Optimized Potential for Liquid Simulations (OPLS) force field. The default Desmond protocol was applied to equilibrate the prepared system and was followed by a 50ns NPT simulation to equilibrate the system. The equilibrated system was saved every 5 ps of time intervals. Potential energy of the entire system was calculated. Stability of the docked complex was evaluated from their root mean square deviation (RMSD) plots. A root mean square fluctuation (RMSF) plot of the backbone atoms of each residue was also created. A snapshot of the protein-peptide complexes were generated using PyMOL (The PyMOL Molecular Graphics System, Version 1.8 Schrödinger, LLC.).

### Surface plasmon resonance (SPR) analysis

Biacore T200 (GE Healthcare, USA) was used for real-time binding interaction studies. Recombinant human TLR4/MD2 (R&D 3146-TM-050) complex protein and MD2 (R&D 1787-MD) protein were purchased from R&D Systems (Minneapolis, USA). For binding analyses, CM5 series chips (GE Healthcare) were activated and the reference flow-cell was blocked by 1 M ethanolamine (pH = 8.5). The ligand protein MD-2 dilution (10 ug/mL in 10 mM Acetate buffer pH 5) or HMGB1 (20 ug/mL in 10 mM acetate buffer pH = 4.5) or TLR4/MD2 complex (10 ug/mL in 10 mM acetate buffer pH 4.5) were subjected to the sample flow-cell at the flow rate of 10 uL/min until the surface Plasmon resonance reached 1000–2000 RU. Analytes (Folic acid analogues) diluted into 5 different concentrations were sequentially injected at a flow rate of 30uL/min for 60s or 120s (TLR4/MD-2). The dissociation time was set for 1 minute or 2 minutes (TLR4/MD-2). The equilibrium dissociation constant (KD) was obtained to evaluate the binding affinity by using the BIAEvaluation 2.0 software (GE Healthcare) supposing a 1:1 binding ratio. At least 3 independent experiments were performed.

For the inhibition assay, wild type HMGB1 (20 ug/mL in 10 mM Acetate buffer pH = 4.5) was immobilized onto a CM5 at a flow rate of 10 uL/min until the surface Plasmon resonance reached 400 RU. MD-2 (0.5 uM) was incubated with or without folic acid analogues (the concentrations were varied (2X) from 10uM to 312.5 nM) for 15 minutes prior to injection of the mixture on the chip at 30 uL/min for 60s. The dissociation time was set for 1 minute. Binding experiments were conducted in 1XPBS + 0.05% Tween 20 as the running buffer, and at least 3 independent experiments were performed.

### Preparation of HMGB1 protein

Recombinant HMGB1 was expressed and purified as described previously [4, 15]. The cytokine-stimulating disulfide HMGB1 was characterized by LC-MS/MS [16]. HMGB1 was extracted with Triton X-114 to remove any contaminating LPS. The LPS content in HMGB1 was measured by the Chromogenic Limulus Amebocyte Lysate Assay (Lonza). The LPS content in HMGB1 protein was < 0.1 EU/mg protein as measured by the limulus assay.

### Cell isolation and culture

Peripheral-blood mononuclear cells were isolated from the blood of normal anonymous volunteers (Long Island Blood Services) over a Ficoll-Hypaque (Pharmacia Biotech) density gradient. Human primary monocytes were isolated by adherence and allowed to differentiate into macrophages for 7 days in complete DMEM medium containing 10% heat-inactivated human AB serum, 100 U/mL penicillin, 100 ug/mL streptomycin, and 1 ng/mL MCSF in 96-well culture plates at 105 cells/well. All experiments were carried out in serum-free Opti-MEM I medium.

### Cytokine measurement

Human primary macrophages in 96-well plates were stimulated with HMGB1 at 1 ug/mL, plus increasing amounts of P5779 (or folic acid analogues) in serum-free Opti-MEM I medium as indicated for 16h. TNF release was measured using an ELISA kit (R&D Systems, Inc) according to the manufacturer instruction.

### Data analysis

For the SPR inhibition assay, analysis of covariance (ANCOVA) was used to model inhibition as a function of log (base 10) concentration and treatment group. Prior to applying ANCOVA, both the normality and homogeneity of slopes assumptions were verified. Upon finding a significant treatment group effect, Dunnett’s test was used to compare each group to P5779. For the cytokine measurement, one-way analysis of variance (ANOVA) was used to compare mean TNF levels among the 10 treatment groups (including HMGB alone serving as the control group). The normality and homogeneity of variance assumptions were verified. Upon finding a significant treatment group effect, Dunnett’s test was used to compare each group to HMGB-1 alone. A result was considered statistically significant if *p* < 0.05.

## Results

### Identification of binding site on TLR4/MD-2

**Fig 1:**
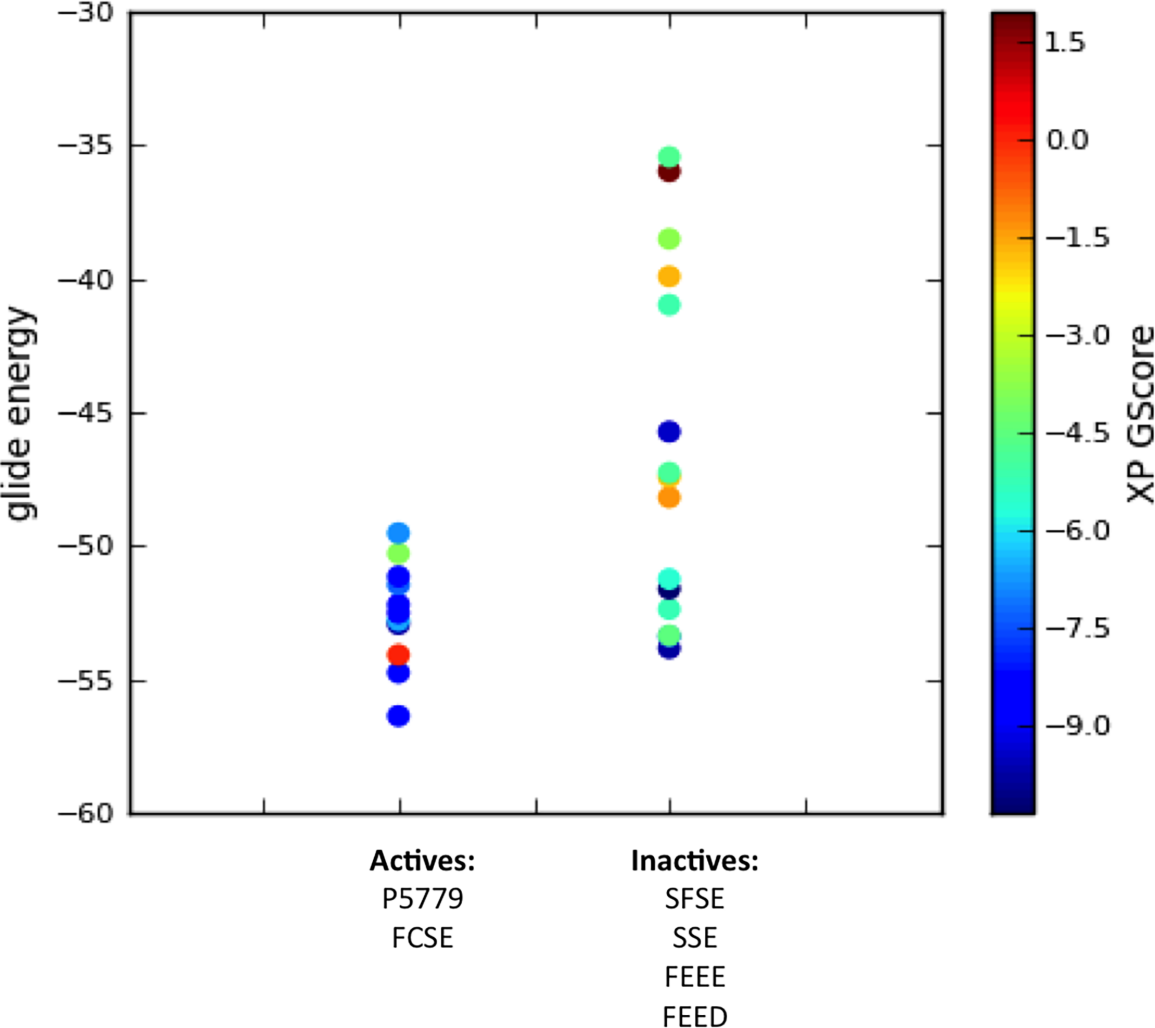
Docking score and glide energy plots of active and inactive tetramers on TLR4/MD-2 complex. A trend correlation of biological activity and docking energy (active compound docks better with lower glide energy) suggests a promising bind site.

In previous studies, we reported a tetramer peptide P5779 (FSSE, the serine homologue of FC^106^SE on HMGB1) directly bound to the TLR4 extracellular adapter MD-2 and inhibited HMGB1-induced TNF release in human macrophages. A series of control trimer or tetramer peptides spanning the Cys106 region, including SFSE, FEED, FEEE, and SSE, showed no binding to MD-2 and were not active *in vitro* [3]. Here, we carried out an extra precision glide docking study with all the active and inactive peptides on the TLR4/MD-2 complex (PDB: 3FXI, chain A and C) to identify an accurate binding site. The lower score/energy indicates a better fit for the protein target (S1 Table). We observed that the active peptides (FCSE and P5779) have lower binding energy and better glide scores compared to the inactive ones (Fig 1). This plot showed a trend of correlation between the calculated binding free energy and bioactivity of these molecules, indicating the defined docking area is likely the binding site for P5779 and active molecules.

### Fingerprint similarity search and structure-based screening for P5779 mimetics

To overcome the weak pharmacokinetic properties of the peptide tetramer P5779 and to enrich the HMGB1 antagonist compound catalog, we carried out a three-step similarity search followed by a structure-based docking method to screen P5779-like small molecules within the Drugbank database (version 4.3). The advantage of combining methods is to capture chemical information in different aspects and reduce the redundancy of artifacts that would be introduced by a single approach.

**Fig 2:**
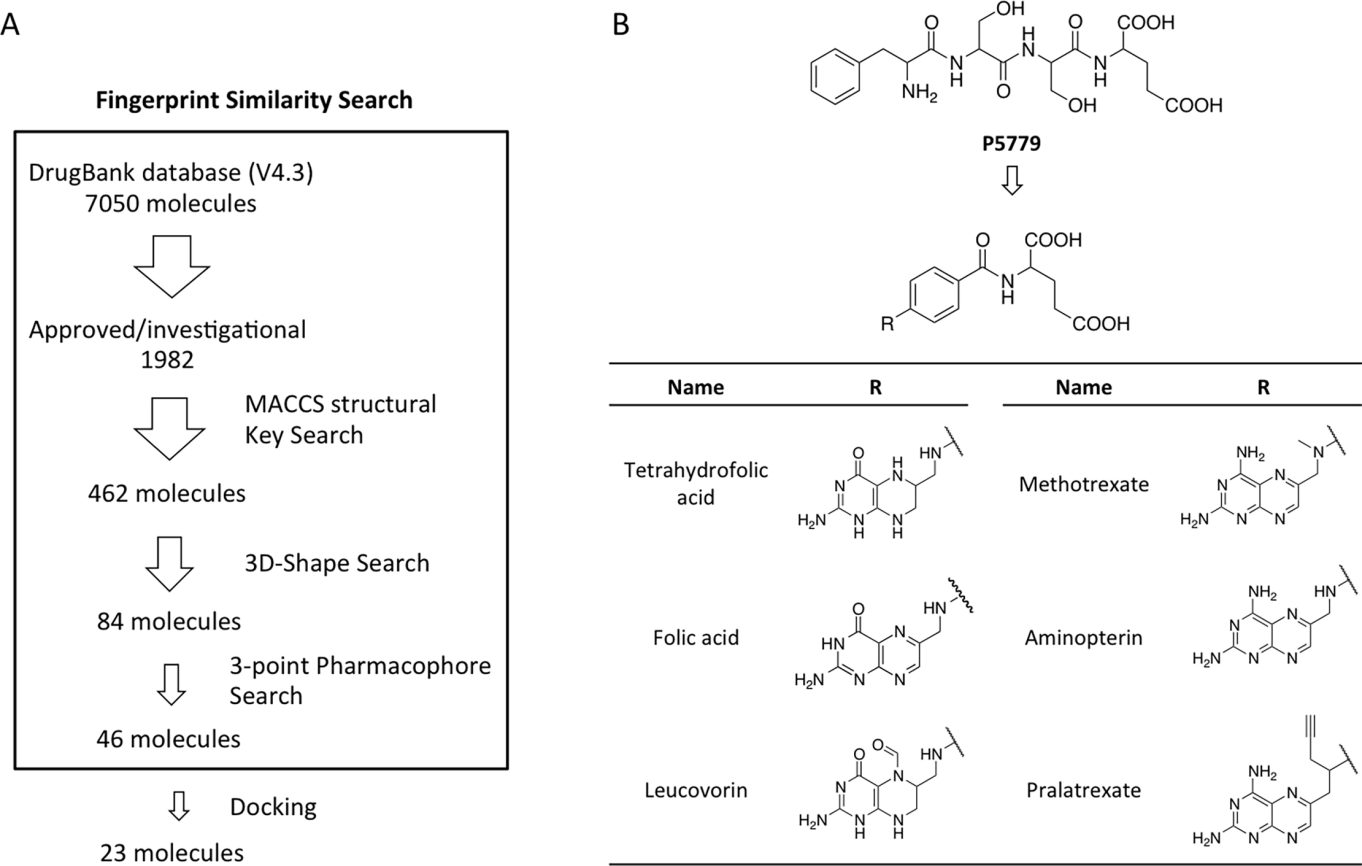
Fingerprint similarity search and structure-based screening for P5779 mimetics. (A) Flowchart of virtual screening. (B) Structures of P5779 and the hits.

The Drugbank database covers 7,050 drug entries, including FDA approved drugs, nutraceuticals, and experimental compounds. A total of 1,982 FDA approved/investigational drugs were submitted to a three-step similarity search using three fingerprint types to examine different aspects of the consensus structure (Fig 2A)[17]. The screening gave 46 potential hits out from the initial 1,982 drugs. These molecules were subsequently docked to the binding site identified on TLR4/MD-2 complex in parallel with P5779 as a reference. Molecules with docking scores ranking below P5779 dock poses were eliminated, which yielded 23 hits. Among these hits, we found the most populated group of 6, tetrahydrofolic acid, folic acid, methotrexate (MTX), pralatrexate, aminopterin and leucovorin, shared a common 2-benzamidosuccinic acid structure and a pteridine ring system (Fig 2B). The structure similarity of the folic acid analogues and P5779 suggested they could potentially display comparable biological effects.

### Molecular dynamic simulation of P5779 and folic acid with TLR4/MD-2

**Fig 3:**
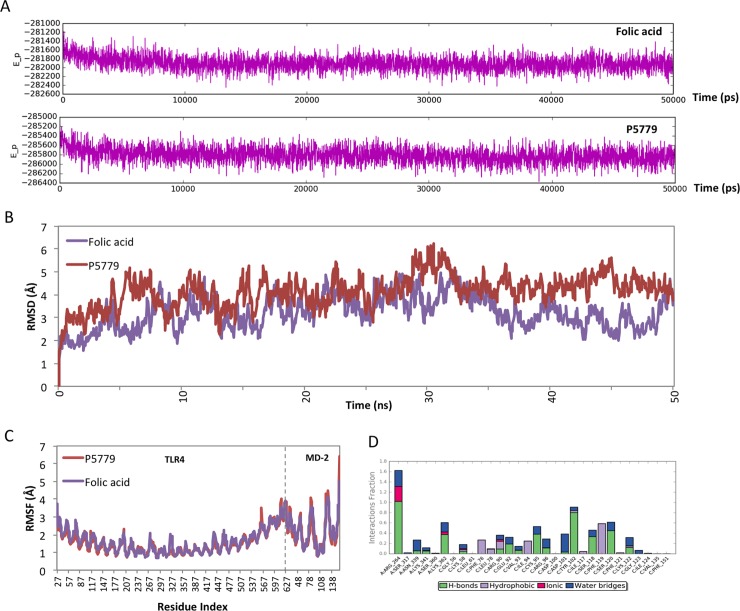
MD simulation trajectory (50 ns) analysis of P5779 and folic acid with TLR4/MD-2 complex. (A) The potential energy plots showing the relatively stable complex system. (B) RMSD plot representing small deviation along the stable region preceded by small rearrangement from the initial conformation. (C) RMSF graph representing the extent of conformational arrangement upon P5779 and folic acid binding to TLR4/MD-2 complex. (D) Protein-ligand contacts during the folic acid/TLR4/MD-2 simulation.

**Fig 4:**
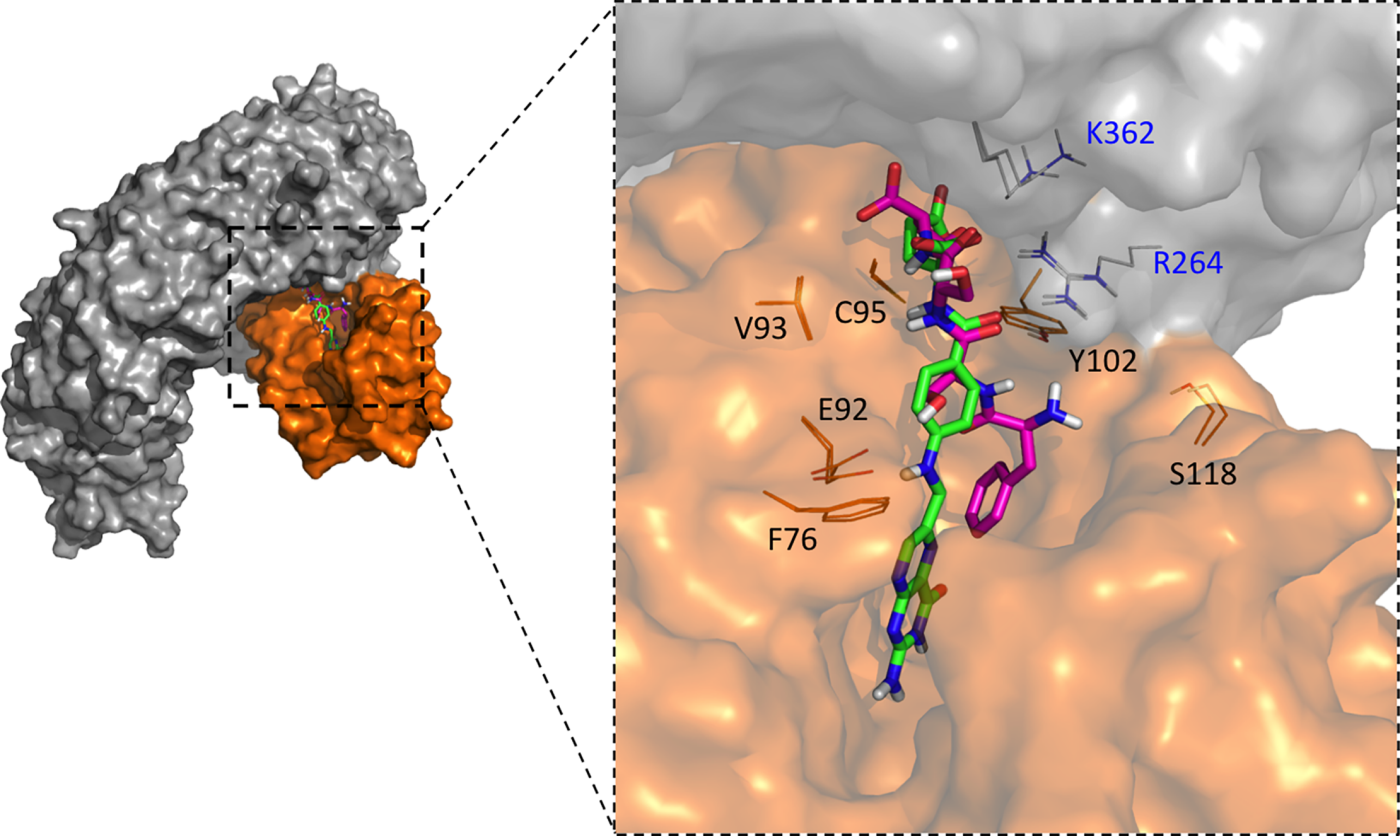
Overlap of folic acid (green) and P5779 (purple) binding poses on TLR4/MD-2.

To exam the dynamic interactions of TLR4/MD-2 complex with P5779 or folic acid, we carried out 50 ns molecular dynamic simulation studies. The potential energy plot of folic acid or P5779 with TLR4/MD-2 (Fig 3A) suggested a well-equilibrated complex system through the simulation. The root mean square deviation (RMSD) during the simulation was analyzed with respect to initial structures in order to provide the structural deviation with respect to time (Fig 3B). At the equalization stage, the small deviation (< 2Å) indicated a stable binding of both molecules to TLR4/MD-2 protein complex. Furthermore, root-mean-square fluctuation (RMSF) of the protein backbone atoms from their time-average position was examined to address the high conformational arrangement area upon ligand binding. The RMSF plot showed higher fluctuations around of MD-2 and suggested that folic acid binding to TLR4/MD-2 has comparable influence on the receptor protein as P5779 binding (Fig 3C). Overlapping the binding poses showed that folic acid binds to TLR4/MD-2 complex in a similar manner as P5779. The glutamic acid group on folic acid binds to R264 and K362 on TLR4. The E92 and Y102 on MD-2 form hydrogen bonding with the amine and carbonyl group on the para-amino benzoic acid region of the folic acid (Fig 4). The protein-ligand contacts plot summarized the interactions throughout the folic acid/TLR4/MD-2 simulation. We found very stable interactions between folic acid and R264, K362 and Y102 (MD-2) during the simulation (Fig 3D).

### Folic acid analogues directly bind to MD-2

**Fig 5:**
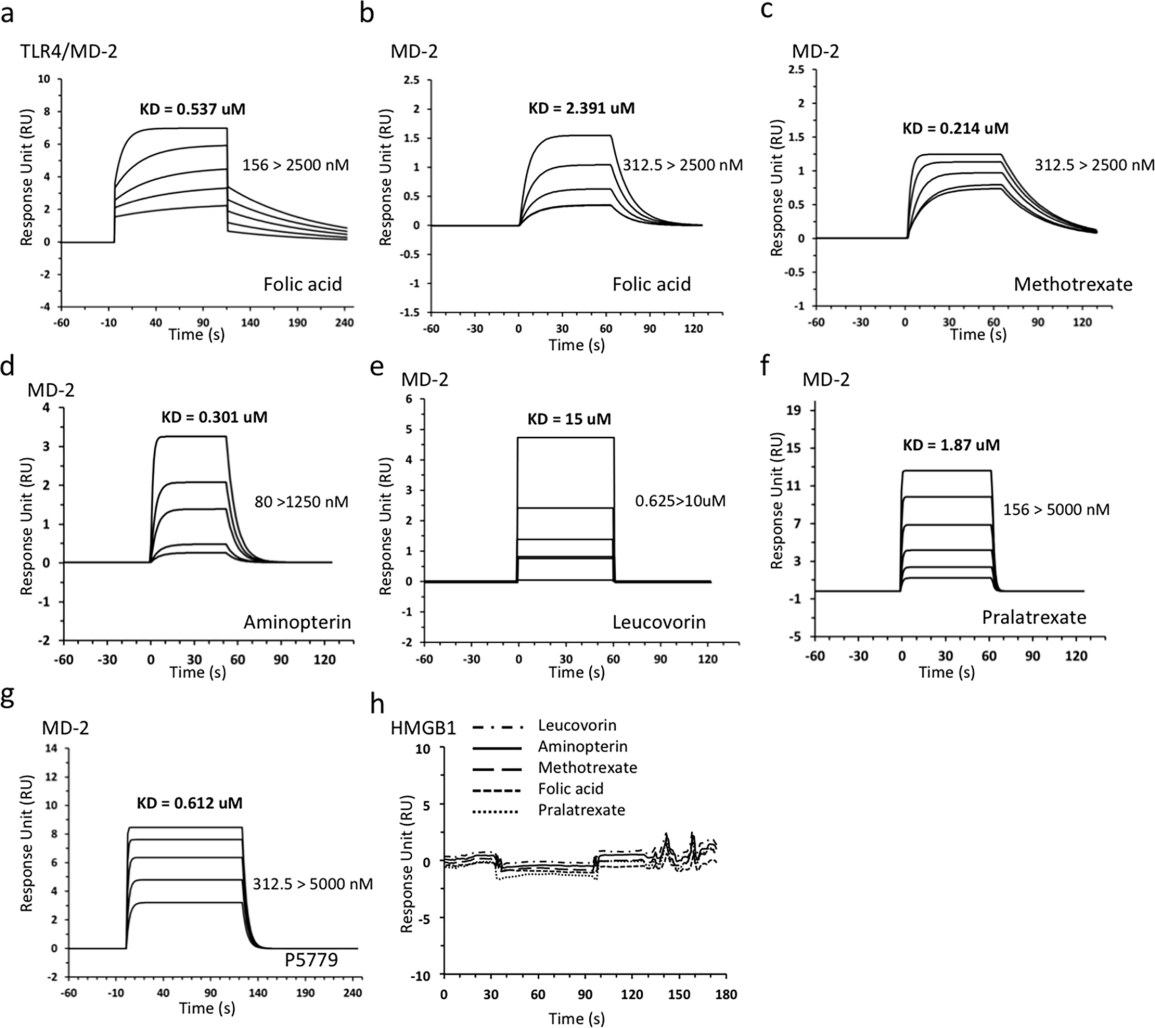
SPR studies of folic acid analogues show direct binding to MD-2 of the TLR/MD-2 complex.

The tetramer peptide P5779 has been identified to bind to MD-2 with a KD value of approximately 0.65 μM and inhibited HMGB1/MD-2 interaction with an IC_50_ of 29 nM [3]. We asked whether the P5779-like folic acid analogues bind to MD-2. Real time surface plasmon resonance (SPR) analysis (Biacore T200) was employed to test the binding activity between folic acid analogues and MD-2. SPR analysis was carried out by immobilizing MD-2 on the CM5 chip as a ligand; analyzing the binding of compounds in gradient concentration as analytes. The KD value was obtained was as a measure of the binding affinity. Tetrahydrofolic acid was excluded in this experiment due to its decomposition over time. P5779 was used as a positive control. Not surprisingly, SPR analysis showed all five folic acid analogues bound directly to MD-2 protein (Fig 5B–5F). Compared to P5779 (Fig 5G), MTX and aminopterin exhibited higher binding affinity to MD-2 with the KD values of 214 nM and 301 nM, respectively (Fig 5C and 5D). Folic acid showed moderate binding to MD-2 (Fig 5B) but higher affinity to the TLR4/MD-2 complex (Fig 5A) suggesting that TLR4 contributes to the binding as well. This supports our observation with the modeling studies that P5779 binds at the TLR4/MD-2 interface. Finally, none of these analogues demonstrated binding to HMGB1 (Fig 5H).

### Folic acid analogues inhibit HMGB1/MD-2 binding

**Fig 6:**
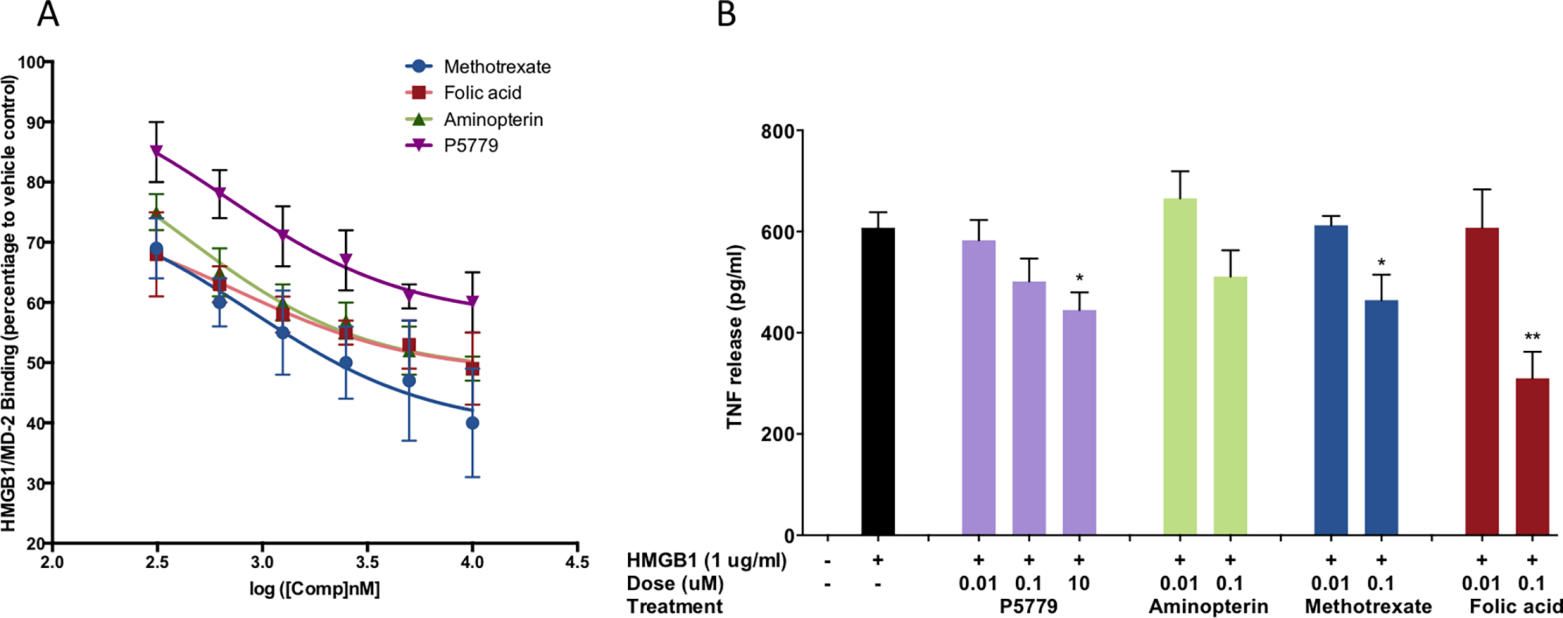
**Folic acid, MTX, and Aminopterin inhibit HMGB1/MD-2 directly binding (A) and suppress HMGB1-induced TNF release in human macrophages (B).** Experimental data are expressed as means ± S.E. for triplicate estimates of individual experiments (*n* = 3). A *p* < 0.05 was considered statistically significant. * Indicates *p* < 0.05; ** indicates *p* < 0.01.

Extracellular HMGB1 activates TLR4 dependent signal by directly binding to TLR4/MD-2. We therefore interrogated whether folic acid and its analogues could interrupt the HMGB1/MD-2 interaction. An SPR inhibitory assay was designed accordingly. We immobilized HMGB1 on the CM5 chip. MTX and aminopterin with good MD-2 binding affinity, as well as the natural vitamin folic acid, were prepared in different concentrations (0–10 uM). Molecules were incubated with 0.5 μM MD-2 for 15 minutes and the mixture was subsequently subjected to SPR analysis as analytes. Inhibitory effect was plotted as the percentage inhibition compared to the control. The folic acid analogues exhibited inhibitory effects against HMGB1/MD-2 binding up to 60% in a concentration-dependent manner (Fig 6A). ANCOVA analysis showed a significant difference between groups with all three groups significantly lower than P5779 (Dunnett’s test: each p<0.0001). Altogether, we demonstrate that the P5779 structurally similar folic acid analogues can act as MD-2 antagonists and inhibit HMGB1/MD-2 binding.

### Folic acid analogues inhibit HMGB1-induced TNF release in human macrophages

To evaluate the therapeutic potential of the folic acid analogues, we studied whether they inhibit HMGB1-induced cytokine production *in vitro*. Human primary macrophages were stimulated with HMGB1 at 1 μg/mL, plus increasing amounts of P5779 (or MTX, or aminopterin, or folic acid) in serum-free Opti-MEM I medium as indicated for 16h. TNF release was measured by ELISA as reported in Fig 6B. Both P5779 and the three folic acid analogues inhibited HMGB1-induced TNF release in primary human macrophages in a dose-responsive manner. Compared to tetrapeptide P5779, the folic acid and MTX exhibit a similar inhibitory effect at a lower concentration.

## Discussion

The peptide tetramer P5779 was identified as an MD-2 antagonist by directly binding to the TLR4 adapter protein MD-2 and was able to consequently disrupHMGB1-TLR4/MD-2 binding and downstream effects (TNF release). P5779 protected mice against hepatic ischemia/reperfusion injury, chemical toxicity and sepsis without interfering with LPS-induced cytokine/chemokine production [3]. Thus P5779 could have a valuable therapeutic impact in attenuating DAMP-mediated inflammation while preserving antimicrobial immune responsiveness. Like most peptide therapeutics, P5779 suffers from poor pharmacokinetic properties. In the current study, an integrated *in silico* virtual screening found a series of P5779-mimetic FDA approved molecules, which retained the critical interactions of P5779 and TLR4/MD-2. These pteroglutamic acid analogues, including MTX, folic acid, pralatrexate, aminopterin and leucovorin, showed direct binding to TLR4/MD-2 and inhibited HMGB1/MD-2 binding in SPR studies. Finally, we establish that the top two analogues (folic acid and MTX), reduce HMGB1-induced TNF release in human macrophages.

High-doses of antifolates, such as MTX, aminopterin, or pralatrexate, act by competitively inhibiting the binding of dihydrofolate reductase (DHFR) to folate, and disrupting *de novo* synthesis of DNA and RNA, thereby retarding the proliferation of cancer cells [18]. Their cytotoxicity’s and side effects, such as inflammation in the digestive tract, hepatotoxicity, pulmonary damage, myelosuppression and nephrotoxicity, are well known. Interestingly, their analogue molecule folic acid has been shown to have anti-inflammatory and hepato-protective effects in an acetaminophen-induced acute liver failure model and to counteract the effect of acetaminophen [19], and is widely given in combination with MTX to decrease the toxicity.

Apparently, the therapeutic effect versus the toxicity of MTX are dose dependent. In addition to its use in cancer, MTX is also widely used for treatment of inflammatory diseases, such as rheumatoid arthritis, SLE, psoriasis and Crohn’s disease for decades, due to its beneficial immunosuppressive and anti-inflammatory effects when administered in low dosage [20]. Although not yet fully understood, it is believed MTX’s low dosage immunosuppressive effect is not through DHFR. Multiple mechanisms appear to be involved in the MTX anti-inflammatory effect, including: the inhibition of adenosine A_*2A*_ receptor (A_*2A*_R) [21]; inhibition of T cell activation; selective down-regulation of B cells; increasing CD95 sensitivity; inhibition of methyltransferase activity [22]; and inhibition of the binding of interleukin 1β to its cell surface receptor (IL-1R) [23].

**Fig 7:**
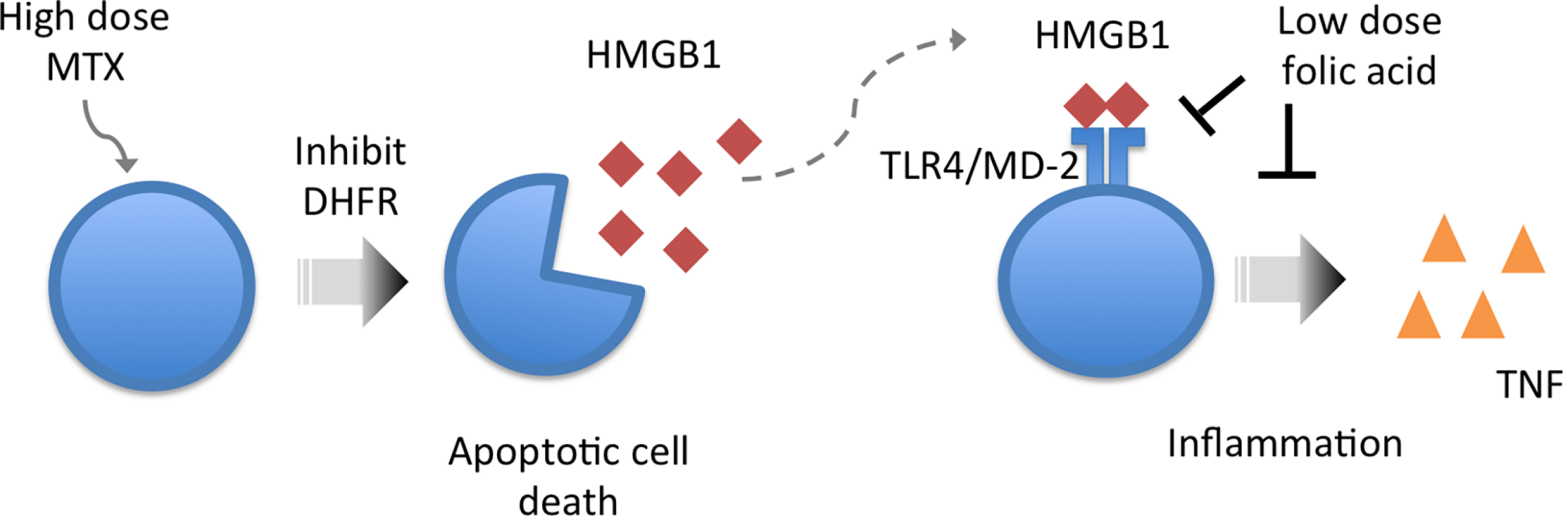
Proposed mechanism of the anti-inflammatory effect of pteroglutamic acid analogues.

Our studies implicate an additional mechanism for the anti-inflammatory effect of the pteroglutamic acid analogues. High dose antifolate MTX and analogs are widely used as chemotherapeutic drugs, which inhibit DNA synthesis and induce apoptosis. During apoptotic cell death, the extracellular HMGB1 can be released [24] and therefore triggers HMGB1-induced inflammation, which can lead to a cascade of inflammatory responses [25]. Our observations support that the pteroglutamic acid analogues, when given in low dosage, seem to regulate HMGB1-induced inflammation through directly binding to TLR4/MD-2 and therefore inhibit HMGB1-induced inflammation (Fig 7). Other research also suggests the folic acid mediates effects through the TLR4 receptor [26]. Interestingly, a recent study hypothesized the anti-inflammatory effect of MTX is due to direct binding to HMGB1 and inhibition of HMGB1-induced TLR4 signaling. Biotinylated MTX (bio-MTX) was shown to bind to HMGB1 in SPR assays [27]. However, the modification on the molecule could interfere with its binding to the protein, although the authors argued that mmobilizing the mixture of both *α* and *β*—biotinylated MTX on the chip in SPR study compensated for the issue [27]. In contrast, our study using non-biotinylated MTX and analogues showed no binding to HMGB1, but tight binding to TLR4/MD-2. The contradictory results reveal that the binding of bio-MTX to HMGB1 might be due to a false binding of the modified molecule. Overall, we conclude that the pteroglutamic acid analogue drugs with improved pharmacokinetic properties are structurally similar to the indirect HMGB1 antagonist peptide P5779. Most noteworthy, folic acid, a B vitamin supplement with minimal toxicity and a natural substrate of DHFR, showed good inhibitory effect on HMGB1-induced TNF release in human macrophages. Furthermore, our studies suggest a new mechanism for the well-known MTX anti-inflammatory effects and are worth additional investigation.

## Supporting information

S1 Table Docking statistics of active and inactive tetramers on the TLR4/MD-2 complex. (PDF)
