# Supplementary material for: Folic acid derived-P5779 mimetics regulate DAMP-mediated inflammation through disruption of HMGB1:TLR4:MD-2 axes
Source: PLoS One. 2018 Feb 15;13(2):e0193028. doi: 10.1371/journal.pone.0193028 (PMC5814057; doi:10.1371/journal.pone.0193028)
Supplement: S1 Table — (PDF) [file pone.0193028.s001.pdf]

	Title	docking score	glide gscore	XP GScore	glide evdw	glide ecoul	glide energy	glide einternal	glide emodel
Active molecules	P5779	0.612	0.065	0.065	-38.514	-15.571	-54.085	6.787	-71.249
	P5779	-3.566	-3.866	-3.866	-34.05	-16.236	-50.286	10.601	-62.563
	P5779	-7.779	-8.326	-8.326	-37.412	-13.737	-51.149	10.323	-74.622
	P5779	-7.936	-8.483	-8.483	-42.095	-14.274	-56.369	15.234	-76.413
	P5779	-8.389	-8.936	-8.936	-35.151	-17.356	-52.507	12.246	-68.883
	P5779	-9.231	-9.779	-9.779	-35.365	-17.567	-52.932	16.506	-70.235
	FCSE	-6.238	-6.538	-6.538	-30.262	-22.539	-52.801	9.062	-69.869
	FCSE	-6.526	-6.826	-6.826	-28.918	-20.608	-49.525	10.123	-67.614
	FCSE	-7.082	-7.382	-7.382	-27.201	-24.257	-51.458	11.656	-70.895
	FCSE	-7.925	-8.473	-8.473	-37.173	-17.578	-54.75	12.352	-70.119
	FCSE	-8.041	-8.588	-8.588	-37.527	-14.679	-52.205	9.253	-69.103
Inactive molecules	FEED	-4.954	-5.213	-5.213	-38.453	-13.92	-52.373	17.095	-64.494
	FEED	-9.749	-10.364	-10.364	-36.276	-15.333	-51.61	17.955	-61.436
	FEEE	-6.882	-7.497	-7.497	-36.353	-17.042	-53.394	13.693	-63.524
	FEEE	-9.124	-9.383	-9.383	-32.829	-12.901	-45.73	15.174	-59.258
	FEEE	-9.223	-9.838	-9.838	-30.668	-23.165	-53.833	11.686	-67.649
	SFSE	-0.677	-1.262	-1.262	-27.94	-20.249	-48.189	7.876	-62.965
	SFSE	-1.21	-1.795	-1.795	-27.237	-20.192	-47.429	10.776	-63.109
	SFSE	-3.907	-4.492	-4.492	-33.772	-19.585	-53.356	11.851	-68.403
	SFSE	-4.537	-4.813	-4.813	-30.916	-16.347	-47.263	11.667	-58.566
	SFSE	-4.976	-5.562	-5.562	-32.094	-19.152	-51.246	14.935	-63.236
	SSE	2.273	1.981	1.981	-26.253	-9.724	-35.977	5.844	-45.833
	SSE	-1.308	-1.6	-1.6	-22.188	-17.727	-39.914	12.417	-45.073
	SSE	-3.474	-3.767	-3.767	-16.694	-21.817	-38.511	8.073	-50.753
	SSE	-4.228	-4.787	-4.787	-17.777	-17.666	-35.442	4.143	-44.915
	SSE	-4.861	-5.154	-5.154	-20.159	-20.819	-40.979	11.792	-47.427

Suppl. Table 1. Docking statistics of active and inactive tetramers on TLR4/MD-2 complex.
